# Dietary Inulin Modulates Intestinal Health and Muscle Nutritional Composition in Juvenile Silver Pomfret (*Pampus argenteus*)

**DOI:** 10.3390/foods15132391

**Published:** 2026-07-05

**Authors:** Cuizhi Zhang, Jiabao Hu, Linying Wang, Zhouji Fang, Suling Sun, Man Zhang, Yongyong Li, Yajun Wang, Lingling Jia

**Affiliations:** 1 College of Food Science and Engineering, Ningbo University, Ningbo 315832, China; 18866812405@163.com (C.Z.); 18835493395@163.com (L.W.); 15968912541@163.com (Z.F.); liyongyong@nbu.edu.cn (Y.L.); 2 College of Marine Sciences, Ningbo University, Ningbo 315832, China; hujiabao@nbu.edu.cn (J.H.); zhangman@nbu.edu.cn (M.Z.); 3 Institute of Quality Safety and Nutrition of Agricultural Products, Zhejiang Academy of Agricultural Sciences, Hangzhou 310021, China; sunsuling123@126.com

**Keywords:** inulin, silver pomfret, muscle composition, amino acids, fatty acids, nutritional quality, gut microbiota, intestinal health

## Abstract

The silver pomfret (*Pampus argenteus*) is a high-value marine food fish, but its aquaculture is limited by juvenile intestinal immaturity, characterized by impaired digestion, barrier dysfunction, and microbial dysbiosis. This study evaluated whether early-life dietary inulin could improve intestinal health and muscle nutritional composition. After an 8-week feeding trial, fish fed a 5 g/kg inulin-supplemented diet showed improved growth performance, as reflected by higher final body weight (+17.2%), WGR (+18.5%), and SGR (+6.4%) than the control group. These benefits were associated with enhanced intestinal morphology, increased α-amylase and lipase activities, upregulated expression of tight junction genes, and a remodeled gut microbiota. These gut-associated changes were accompanied by improved selected muscle compositional traits. Specifically, inulin supplementation enriched essential amino acids, including methionine and threonine, as well as flavor-related amino acids, such as glutamate, glycine, and serine. Meanwhile, the muscle lipid profile was also modified, as reflected by reduced levels of selected saturated fatty acids and increased levels of monounsaturated fatty acids, particularly oleic acid. Collectively, our findings suggest that early-life dietary supplementation with 5 g/kg inulin may support intestinal homeostasis-related indicators and improve selected muscle nutritional traits in farmed silver pomfret.

## 1. Introduction

The silver pomfret (*Pampus argenteus*) is a commercially prized marine fish in Asia, renowned for its tender flesh, few intermuscular bones, and favorable nutritional characteristics, making it a high-value food product in the seafood market [1]. Recent studies and fishery information further highlight its economic and industrial relevance. Silver pomfret has been described as an economically important mariculture species and as one of the highly marketed fish species in the aquaculture industries of Kuwait, India, and China [2]. Regional fishery research has also reported that silver pomfret is among the commonly caught demersal fish groups in Asian coastal fisheries; for example, in Cirebon District, West Java, Indonesia, the average catch was approximately 937.50 tonnes per year, with an average fishing effort of 16,100 trips per year [3]. Owing to high consumer demand and long-standing dependence on wild capture, natural stocks of silver pomfret have been increasingly pressured, highlighting the need to develop reliable aquaculture production for this species [4]. Over the past two decades, our team has established a successful breeding system that now produces nearly one million juveniles annually [5]. Although significant progress has been made in captive breeding [5], industrial-scale farming of silver pomfret is primarily constrained by high juvenile mortality due to growth retardation and compromised immunity, stemming from inherent physiological immaturity of the digestive and immune systems [6,7]. This vulnerability is centered on the intestine, which in young fish is characterized by short villi, low digestive enzyme activity, and limited nutrient absorption capacity [8,9]. More severely, juvenile silver pomfret exhibit a dysfunctional gut ecosystem characterized by microbial instability, reduced abundance of taxa associated with intestinal homeostasis, and a leaky intestinal barrier, which may facilitate pathogen infiltration, translocation, and recurrent infections [10,11]. These physiological limitations not only hinder growth and survival but also potentially compromise the consistency and quality of the final aquaculture product. Therefore, there is an urgent need for targeted nutritional strategies that support intestinal development and overall resilience in early life stages, which is crucial for both sustainable production and ensuring high-quality yield.

Inulin, a natural fructan-type dietary fiber and prebiotic, has been reported to be fermented by intestinal microbiota into short-chain fatty acids (SCFAs), including butyrate, which may contribute to gut health, barrier integrity, and systemic immunity [12,13,14]. Compared to other prebiotics, inulin has been reported to support intestinal development, barrier integrity, microbiota modulation, and immune-related responses in aquatic animals [15]. Accumulating evidence across diverse aquatic species (e.g., *Nibea coibor*, *Totoaba macdonaldi*, and *Ctenopharyngodon idella*) demonstrates that dietary inulin supplementation improves growth performance by enhancing digestive enzyme activities (e.g., α-amylase and lipase), promotes intestinal morphological development (e.g., villus height and integrity), strengthens barrier function via upregulation of tight junction proteins (e.g., *Occludin* and *Claudin-4*), and modulates microbial community structure by altering the abundance of taxa previously associated with probiotic potential, fiber utilization, or opportunistic bacterial groups [16,17]. Beyond supporting basic physiological functions, the modulation of gut homeostasis by prebiotics, such as inulin, may have downstream effects on host metabolism and nutrient partitioning, potentially influencing the composition of edible tissues such as muscle [18]. Previous studies in aquatic animals have also suggested that dietary prebiotics may influence edible muscle quality-related traits, including amino acid composition, fatty acid-related components, nutrient deposition, texture-related traits, and antioxidant status [19]. For example, dietary supplementation with galacto-oligosaccharides and xylo-oligosaccharides has been reported to improve flesh quality and nutritional value in Nile tilapia fed a high-carbohydrate diet [20]. Importantly, the established safety profile of inulin, affirmed by major food safety authorities (e.g., U.S. FDA, EU EFSA), underscores its reliability as a functional ingredient with high translational potential.

Despite the documented benefits of inulin in other aquatic species, its potential to address the multifaceted physiological constraints (immature digestive systems, immune deficiencies, dysbiotic microbiota, leaky gut barriers, and high mortality) of juvenile silver pomfret and, critically, its subsequent impact on muscle nutritional composition, remains largely unexplored. The early-life stage presents a critical window for nutritional intervention, during which gut health may influence not only survival and growth but also nutrient utilization and tissue deposition. Therefore, we speculate that early-life dietary supplementation with inulin may enhance intestinal homeostasis, which in turn would systemically improve growth, health, and ultimately, selected amino acid and fatty acid compositional traits of silver pomfret muscle. This study was designed to systematically evaluate the effects of dietary inulin on the growth, intestinal health, and systemic physiology of silver pomfret, with a particular focus on food science, specifically the detailed amino acid and fatty acid profile of the dorsal muscle as the consumable product. We selected a dietary inclusion level of 5 g/kg based on efficacy reports in other aquatic species [21,22], aiming to test whether this selected inulin level could serve as a gut-targeted nutritional strategy to mitigate production bottlenecks and improve selected nutritional compositional indicators of farmed silver pomfret.

## 2. Materials and Methods

### 2.1. Ethics Statement

All fish experiments were conducted in accordance with the National Institutes of Health Guide for the Care and Use of Laboratory Animals. The protocols were approved by the Animal Care and Use Committee of Ningbo University (NBU20220079).

### 2.2. Experimental Diets

Inulin (≥98% purity, white powder) used in this study was purchased from Baishun Biotech (Xuzhou, China). Two experimental diets were prepared: a control diet and an inulin-supplemented diet. For both diets, the commercial compound feed (Love Larva, Hayashikane Sangyo Co., Ltd., Shimonoseki, No. 3–6, Japan) was crushed and passed through a 60-mesh sieve. For the inulin-supplemented diet, 5 g of inulin was thoroughly mixed with 995 g of powdered commercial feed to prepare 1 kg of final diet, corresponding to an inclusion level of 5 g/kg diet. The control diet consisted of powdered commercial feed alone and was processed identically. Distilled water was gradually added to both diets as needed, and the mixtures were thoroughly mixed by hand until homogeneous. The mixtures were then extruded into pellets using a manual pelletizer (YK60, Shanghai Tianhe Pharmaceutical Machinery Co., Ltd., Shanghai, China), and the pellet diameters were adjusted to 1.00, 1.50, and 2.00 mm according to the mouth size of juvenile silver pomfret. Thus, the control and inulin-supplemented diets underwent the same processing procedures, except for the 0.5% replacement of commercial feed with inulin. After pelletizing, the pellets were dried in a hot-air oven (DHG-9140A, Alphavita Bio-Scientific Dalian, Liaoning, China) at 50 °C until fully dried and then stored at −20 °C until use. The complete feed formulation and proximate nutritional composition are detailed in Table 1.

### 2.3. Experimental Design and Culture Conditions

Juvenile silver pomfret (*Pampus argenteus*) were obtained from a commercial hatchery in Xiangshan Bay, Zhejiang, China. The feeding trial was conducted at the pilot-scale aquaculture facility of Meishan Campus, Ningbo University, Ningbo, China. After a 2-week acclimation period, apparently healthy fish with comparable body size were selected for the feeding trial. A total of 1200 fish with an initial body weight of 1.32 ± 0.63 g were randomly distributed into six 2000 L tanks, with 200 fish per tank. Each dietary treatment was randomly assigned at the tank level to three independent replicate tanks (*n* = 3). A recirculating aquaculture system was used to maintain water quality and oxygen supply under the same culture conditions across all tanks. During the 8-week feeding trial, the water temperature was maintained at 24.0 ± 1.0 °C, dissolved oxygen was kept above 7.0 mg/L, and salinity was maintained at 25.0 ± 1.0‰. Fish were fed the experimental diets three times daily at 08:00, 14:00, and 21:00, at a total daily feeding rate of approximately 10% of the estimated biomass. The amount of feed offered was recorded daily. Tank bottoms were cleaned daily, and approximately 50% of the water volume was replaced. Mortality was monitored and recorded daily, and dead fish were removed immediately.

### 2.4. Sampling

At the end of the 8-week feeding trial, fish were fasted for 24 h and anesthetized with MS-222 at 60 mg/L before sampling. All surviving fish in each tank were counted and batch-weighed for growth performance analysis. Thirty fish were randomly selected from each tank for individual measurements of body weight and body length. For serum biochemical and tissue enzyme activity analyses, six fish were randomly collected from each tank. Blood samples were collected, kept at 4 °C for 4 h, and centrifuged at 4000 rpm for 20 min at 4 °C. The resulting serum was stored at −80 °C until biochemical analysis. The liver and intestine from the same fish were excised, immediately frozen in liquid nitrogen, and stored at −80 °C for enzyme activity assays. For qPCR analysis, intestinal tissues from five fish per tank were preserved in RNA Storage Reagent (Yali, Suzhou, China) overnight at 4 °C and then transferred to −80 °C until RNA extraction. For gut microbiota analysis, intestinal contents from three fish per tank were collected individually, frozen in liquid nitrogen, and stored at −80 °C for 16S rRNA sequencing. For histological analysis, intestinal segments from six fish per tank were randomly collected and fixed in 4% paraformaldehyde. For muscle composition analysis, dorsal muscle samples from three fish per tank were collected and pooled within each tank, immediately frozen in liquid nitrogen, and stored at −80 °C until amino acid and fatty acid analyses.

### 2.5. Scanning Electron Microscopy (SEM) Analysis

The surface morphology of inulin was examined using SEM. Inulin powder was uniformly dispersed and mounted onto an aluminum sample stub using conductive adhesive tape, and loosely attached particles were gently removed using a gentle stream of air to minimize charging and sample drift. The mounted samples were subsequently sputter-coated with a thin layer of gold to enhance electrical conductivity and reduce charging effects induced by electron beam irradiation. After coating, the samples were placed in the SEM chamber and observed at an accelerating voltage of 3 kV and a working distance of 8.0 mm using an S-4800 scanning electron microscope (Hitachi, Tokyo, Japan). Micrographs were acquired at magnifications of 50×, 300×, and 500× to evaluate the overall morphology, aggregation state, and surface structural features of inulin particles.

### 2.6. Nuclear Magnetic Resonance (NMR) Spectroscopy Analysis

1D (^1^H, ^13^C) and 2D (HMQC, COSY) NMR spectra of inulin were recorded on a Bruker AVANCE III HD 600 MHz spectrometer (Bruker Group, Ettlingen, Germany) equipped with a Prodigy BBO cryoprobe at 25 °C. For NMR analysis, 30 mg of inulin was dissolved in 0.6 mL of DMSO-d6, and the resulting solution was transferred into a 5 mm NMR tube for spectral acquisition. All spectra were subsequently processed and analyzed using Mnova NMR software (Version 15.0.0, Mestrelab, Santiago de Compostela, Spain).

### 2.7. Fourier-Transform Infrared Spectroscopy (FTIR) Analysis

FTIR spectra of inulin were obtained using a Fourier-transform infrared spectrometer (Thermo Fisher Scientific, Waltham, MA, USA) at room temperature. For FTIR measurement, an appropriate amount of dried inulin was mixed with KBr powder, finely ground, and compressed into pellets. Spectra were recorded in the range of 4500 to 500 cm^−1^ at a resolution of 4 cm^−1^ and 32 scans. The obtained spectra were further processed and analyzed using Origin software (Version 2018, OriginLab Corporation, Northampton, MA, USA).

### 2.8. Growth Performance Analysis

At the end of the 8-week feeding experiment, silver pomfret were fasted for 24 h to ensure gut clearance. The fish were then anesthetized with MS-222 (60 mg/L). All surviving fish in each tank were counted and batch-weighed to calculate growth performance. Thirty fish per tank were individually measured for body weight and body length to calculate condition factor:

Weight gain rate (WGR, %) = [(final weight − initial weight)/initial weight] × 100

Specific growth rate (SGR, %/d) = [(ln (final weight) − ln (initial weight))/d] × 100

Feed conversion ratio (FCR) = dry diet fed (g)/wet weight gain (g)

Condition factor (CF) = [body weight (g)/body length (cm)^3^] × 100

Hepatosomatic index (HSI, %) = (liver weight/body weight) × 100

Survival rate (%) = (final fish number/initial fish number) × 100

### 2.9. Amino Acid Analysis

Amino acid composition was determined according to the method described [23], with slight modifications. Briefly, 100 mg of pooled dorsal muscle tissue from each replicate was accurately weighed and placed into a 50 mL hydrolysis ampoule containing 10 mL of 6 M HCl. The ampoule was flushed with nitrogen to minimize oxidation and then sealed under vacuum. The samples were hydrolyzed at 110 °C for 22 h in a drying oven. After cooling to room temperature, the hydrolysate was diluted with deionized water and transferred to a rotary evaporator flask to remove excess HCl under reduced pressure. The residue was then redissolved in 0.02 M HCl and adjusted to a final volume of 10 mL. The solution was filtered through a 0.22 μm membrane filter before analysis. Amino acid composition was analyzed using an automatic amino acid analyzer (S-433D, Sykam, Eresing, Germany). Amino acids were identified and quantified by comparison with standard amino acid solutions. Data are expressed as g/100 g wet weight of dorsal muscle. Total essential amino acids (TEAA) and total amino acids (TAA) were calculated as the sum of the corresponding detected amino acids.

### 2.10. Fatty Acid Analysis

Fatty acids were determined according to the method described by Chen et al. [23], with slight modifications. Briefly, 3.0 g of pooled muscle tissue from each replicate was hydrolyzed with 8.3 M HCl, and lipids were extracted using petroleum ether and diethyl ether (1:1, *v*/*v*). The upper ether phase was collected after settling for 10 min, and solvents were removed under reduced pressure. The extracted lipids were then converted to fatty acid methyl esters (FAMEs) via saponification with 8 mL of 2% sodium hydroxide in methanol and refluxing at 80 °C until no oil droplets remained, followed by methylation with 7 mL of 15% boron trifluoride in methanol for 2 min at 80 °C. After cooling, FAMEs were extracted with 3 mL of n-heptane, and the organic layer was dried over anhydrous sodium sulfate before being transferred to an autosampler vial. FAMEs were analyzed using a gas chromatograph (Trace GC Ultra, Thermo Fisher Scientific, USA) equipped with an HB-88 capillary column (100 m × 0.25 mm × 0.20 μm). Hydrogen was the carrier gas (1.3 mL/min, split ratio 5:1). The oven temperature program was as follows: 100 °C for 15 min, increased to 180 °C at 10 °C/min (held 6 min), then to 210 °C at 2 °C/min (held 6 min), and finally to 240 °C at 4 °C/min (held 10 min). Fatty acids were quantified using the external standard method, and their relative percentages were calculated by normalization.

### 2.11. Serum Biochemical Assays

Serum biochemical and immune-related parameters, including glucose (GLU), total cholesterol (T-CHO), triglyceride (TG), acid phosphatase (ACP), and lysozyme (LZM), were measured using commercial assay kits purchased from the Nanjing Jiancheng Bioengineering Institute (Nanjing, China) and Suzhou Grace Biotechnology Co., Ltd. (Suzhou, China). All assays followed manufacturer protocols and were quantified spectrophotometrically using a Varioskan LUX microplate reader (Thermo Fisher Scientific, USA).

### 2.12. Enzyme Activity Analysis in the Liver and Intestine

To prepare enzyme extracts, tissues were homogenized in ice-cold phosphate buffer (1:9, *w*/*v*) and centrifuged at 12,000 rpm for 20 min at 4 °C, and the supernatant was collected for analysis. Antioxidant-related parameters, including superoxide dismutase (SOD), total antioxidant capacity (T-AOC), catalase (CAT), glutathione peroxidase (GPX), and malondialdehyde (MDA), were measured using commercial assay kits purchased from Beyotime Biotechnology Co., Ltd. (Shanghai, China), the Nanjing Jiancheng Bioengineering Institute (Nanjing, China), and Suzhou Grace Biotechnology Co., Ltd. (Suzhou, China). Digestive enzyme activities, including α-amylase, lipase, and pepsin, were determined using commercial assay kits purchased from the Nanjing Jiancheng Bioengineering Institute (Nanjing, China). Total protein content was measured by the bicinchoninic acid (BCA) assay to normalize enzyme activities. All assays followed the manufacturer’s instructions and were quantified with a Varioskan LUX microplate reader (Thermo Fisher Scientific, USA).

### 2.13. Intestinal Histological Examination

Portions of the mid-intestine from six fish per tank were dissected and fixed in 4% paraformaldehyde for 24 h. The samples were dehydrated through a graded ethanol series, embedded in paraffin, sectioned into 5-μm-thick slices, and stained with hematoxylin and eosin (H&E). The stained sections were sealed and scanned using a KF-PRO-005-EX digital pathology slide scanner (Ningbo Jiangfeng Biological Technology Co., Ltd., Ningbo, China). Histological parameters, including villus height, villus width, muscle thickness, and goblet cell count, were analyzed based on the scanned images using ImageJ v1.49 software.

### 2.14. Quantitative Real-Time PCR for Intestinal Gene Expression

Total RNA was extracted from intestinal tissue using TRIzol™ reagent (Vazyme Biotech Co., Ltd., Nanjing, China) according to the manufacturer’s instructions. RNA quality was assessed by 1% agarose gel electrophoresis and a NanoPhotometer^®^ spectrophotometer (IMPLEN, Westlake Village, CA, USA), while RNA concentration was measured using the Qubit^®^ RNA Assay Kit and Qubit^®^ 2.0 Fluorometer (Life Technologies, Carlsbad, CA, USA).

The extracted RNA (1 μg) was reverse-transcribed into complementary DNA (cDNA) using the HiScript^®^ III RT SuperMix (R333, Vazyme, Nanjing, China) following the manufacturer’s protocol. Quantitative real-time PCR (RT-qPCR) was performed in a 10 μL reaction system containing 5 μL SYBR Green PCR Master Mix (Vazyme, China), 3.2 μL double-distilled water (ddH_2_O), 0.4 μL forward primer (10 μM), 0.4 μL reverse primer (10 μM), and 1 μL cDNA template. Amplification was conducted on an Eppendorf Mastercycler ep Realplex PCR system (Hamburg, Germany) under the following cycling conditions: initial denaturation at 95 °C for 30 s, followed by 40 cycles of 95 °C for 10 s and 60 °C for 30 s. A melting curve analysis was performed from 60 °C to 95 °C, increasing by 0.5 °C every 5 s, to verify the specificity of the amplified products. Primers were designed using Primer Premier 5.0 software (Premier Biosoft International, Palo Alto, CA, USA) (Table 2). To ensure reliable normalization, β-actin was selected as the internal control due to its stable expression and amplification efficiency. Relative expression levels were calculated using the 2^−ΔΔCT^ method [24]. The expression stability of β-actin across samples is shown in Table S1.

### 2.15. Intestinal Microbial DNA Extraction and Full-Length 16S rRNA Sequencing in Young Silver Pomfret

Microbial DNA was extracted from the intestinal contents of silver pomfret using the E.Z.N.A.^®^ DNA Stool Kit (Omega Bio-Tek, Norcross, GA, USA), following the manufacturer’s protocols. The full-length 16S rRNA gene (V1–V9 region) was amplified using polymerase chain reaction (PCR) with the primers 27F (5′-AGRGTTYGATYMTGGCTCAG-3′) and 1492R (5′-RGYTACCTTGTTACGACTT-3′), each containing an eight-base barcode unique to each sample. PCR amplification was performed in triplicate in a 20 μL reaction mixture containing 4 μL of 5× FastPfu Buffer, 2 μL of 2.5 mM dNTPs, 0.8 μL of each primer (5 μM), 0.4 μL of FastPfu Polymerase, and 10 ng of template DNA. The cycling conditions were as follows: initial denaturation at 95 °C for 2 min, followed by 27 cycles of 95 °C for 30 s, 55 °C for 30 s, and 72 °C for 60 s, with a final extension at 72 °C for 5 min. The PCR products were extracted from a 2% agarose gel and purified using the AxyPrep DNA Gel Extraction Kit (Axygen Biosciences, Union City, CA, USA), according to the manufacturer’s instructions.

Library preparation was performed using blunt-end ligation, following the manufacturer’s protocol (Pacific Biosciences, Menlo Park, CA, USA). Amplicon sequencing was conducted by Shanghai Biozeron Biotechnology Co., Ltd. (Shanghai, China) on a PacBio Sequel II 8M platform using Sequencing Kit 2.0 chemistry. PacBio raw reads were processed using SMRT Link Analysis software (version 9.0) to obtain demultiplexed circular consensus sequence (CCS) reads with the following settings: minimum number of passes = 3 and minimum predicted accuracy = 0.99. Raw reads were filtered by sequence length and quality using SMRT Portal (version 2.3.0). Barcode and primer sequences were trimmed from the reads, and low-quality reads containing 10 or more consecutive identical bases were discarded. Chimeric sequences were then detected and removed using UCHIME (version 4.2.40). Sequencing depth was evaluated based on the number of high-quality CCS reads retained after quality filtering. Before downstream analyses, the OTU table was normalized by rarefaction to an equal sequencing depth of 26,996 reads per sample. High-quality reads were clustered into operational taxonomic units (OTUs) at a 98.65% similarity threshold using UPARSE (version 7.1). Taxonomic classification was performed using the RDP Classifier against the SILVA (SSU132) 16S rRNA database with a confidence threshold of 70% [25].

### 2.16. Statistical Analysis

Each dietary treatment included three replicate tanks, and the tank was considered the experimental unit for all formal statistical analyses. To avoid pseudoreplication, individual fish sampled from the same tank were treated as subsamples rather than independent experimental units. For variables measured from multiple fish within the same tank, individual fish-level values were first averaged within each tank, and the resulting tank-level means were used for statistical comparison. Thus, unless otherwise stated, statistical analyses were performed using *n* = 3 replicate tanks per treatment. Data are presented as mean ± standard deviation (SD). Differences between the control and inulin-supplemented group were analyzed using an unpaired two-tailed Student’s *t*-test in GraphPad Prism 8.0 software (San Diego, CA, USA), with *p* < 0.05 considered statistically significant.

Beta-diversity was evaluated based on Bray–Curtis dissimilarity calculated from OTU relative abundance profiles. Non-metric multidimensional scaling (NMDS) was used to visualize differences in overall gut microbial community structure between the control and inulin-supplemented group. The reliability of the NMDS ordination was evaluated using the stress value. For taxonomic comparisons, the relative abundances of selected dominant taxa at the phylum, family, genus, and species levels were calculated from the taxonomically assigned OTU table and compared using the same tank-level statistical framework.

Kaplan–Meier survival curves were generated to descriptively visualize survival patterns during the 56-day feeding trial and are presented as Figure S1, while final survival rates calculated for each tank were used for statistical comparison.

## 3. Results

### 3.1. Morphological and Structural Characterization of Inulin

**Figure 1:**
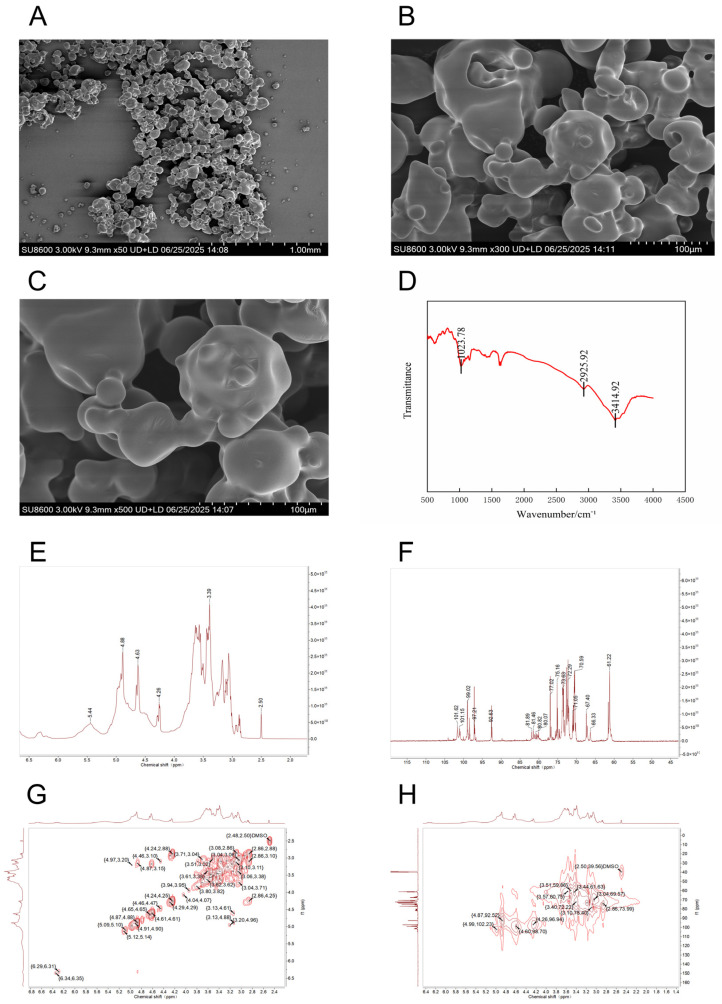
Morphological and structural characterization of inulin. (**A**–**C**) Scanning electron microscopy images of the inulin sample at magnifications of 50×, 300× and 500×. Scale bars: 1.00 mm in (**A**) and 100 μm in (**B**,**C**). (**D**) FTIR spectrum of the sample. (**E**) ^1^H NMR spectrum and (**F**) ^13^C NMR spectrum of the sample. (**G**,**H**) Two-dimensional NMR spectra, including COSY and HMQC.

The structural characteristics of inulin were investigated by SEM, FTIR spectroscopy, and NMR spectroscopy. SEM images showed that inulin particles exhibited irregular spherical morphology with a loose, porous, and aggregated structure across different magnifications (Figure 1A–C). The FTIR spectrum displayed the characteristic absorption bands of inulin (Figure 1D), including a broad O–H stretching band at 3415 cm^−1^, a C–H stretching band at 2926 cm^−1^, and a strong absorption at 1024 cm^−1^ corresponding to C–O and C–O–C stretching vibrations, confirming the presence of a fructan backbone. The 1D and 2D NMR spectra further revealed typical inulin structural features (Figure 1E–H). In the ^1^H NMR spectrum, signals were mainly distributed at δ 3.0–5.3 ppm, with overlapping peaks at δ 3.2–4.2 ppm assigned to the ring protons of fructofuranosyl residues. In the ^13^C NMR spectrum, the characteristic signals at δ 98–101, 61–62, and 67–81 ppm were assigned to C–2, C–1/C–6, and C–3, C–4, and C–5 signals of fructose residues, respectively. In addition, the COSY and HMQC spectra showed proton-proton and proton-carbon correlations consistent with fructofuranosyl units. Collectively, these analyses demonstrated that the sample possessed the typical inulin structure, characterized by a backbone of β-(2 → 1)-linked fructosyl units, confirming the presence of β-(2 → 1) glycosidic bonds as the primary structural feature.

### 3.2. Dietary Inulin Supplementation Enhances Growth Performance in Silver Pomfret

The effects of dietary inulin supplementation on young silver pomfret were evaluated, with a focus on growth performance and feed efficiency (Table 3). Compared with the control group, fish in the inulin group exhibited a 17.2% increase in final body weight, an 18.5% increase in WGR, and a 6.4% improvement in SGR. FCR was lower in the inulin group than in the control group. CF was also significantly higher in the inulin group. Meanwhile, no significant differences were observed in the HSI between the inulin and control groups. Survival patterns during the feeding trial are shown in Figure S1 by Kaplan–Meier analysis.

### 3.3. Dietary Inulin Alters Selected Muscle Amino Acid Profile in Silver Pomfret

Dietary inulin supplementation altered the contents of essential and flavor-related amino acids in the dorsal muscle of juvenile silver pomfret (Table 4). Compared with the control, the inulin group exhibited higher levels of methionine (Met), threonine (Thr), histidine (His), and total essential amino acids (TEAA). Moreover, inulin supplementation also increased the levels of several flavor-related AAs, including glutamic acid (Glu), glycine (Gly), and serine (Ser). The total amino acid (TAA) content was also higher in the muscle of the inulin group compared to the control group. Collectively, these results indicate that dietary inulin supplementation was associated with changes in the amino acid profile of silver pomfret muscle.

### 3.4. Dietary Inulin Supplementation Alters Selected Muscle Fatty Acid Composition in Silver Pomfret

Dietary inulin supplementation was associated with changes in selected fatty acid components in the dorsal muscle of juvenile silver pomfret (Table 5). Compared with the control group, the inulin-fed group showed lower levels of specific saturated fatty acids (SFAs), including myristic acid (C14:0), pentadecanoic acid (C15:0), and heptadecanoic acid (C17:0). Concurrently, the inulin-fed group exhibited higher levels of monounsaturated fatty acids (MUFAs), mainly reflected by increased heptadecenoic acid (C17:1) and oleic acid (C18:1n-9) levels. These results indicate that dietary inulin supplementation may influence selected components of the muscle fatty acid profile in juvenile silver pomfret.

### 3.5. Dietary Inulin Improves Serum Metabolism and Enhances Innate Immunity in Juvenile Silver Pomfret

Dietary inulin supplementation modulated selected serum biochemical and innate immune-related parameters in juvenile silver pomfret (Table 6). Specifically, serum GLU levels were significantly elevated in the inulin group. Concurrently, inulin administration resulted in a significant reduction in circulating TG levels. The T-CHO level remained unaffected across the dietary groups. In addition, inulin supplementation significantly enhanced the activities of the innate immune-related enzymes LZM and ACP. Collectively, these results indicate that dietary inulin supplementation altered selected serum metabolic and immune-related indicators in juvenile silver pomfret.

### 3.6. Dietary Inulin Enhances Liver Antioxidant Capacity in Silver Pomfret

Dietary inulin supplementation altered selected hepatic antioxidant-related parameters in juvenile silver pomfret (Table 7). The inulin-fed group exhibited higher T-AOC than the control group. Specifically, the activities of CAT and GPX were significantly elevated in the inulin group. In contrast, no significant differences were observed in SOD activity or MDA level between the two groups. Collectively, these results indicate that dietary inulin supplementation affected selected hepatic antioxidant-related indicators in juvenile silver pomfret.

### 3.7. Dietary Inulin Enhances Intestinal Antioxidant Capacity in Silver Pomfret

Dietary inulin supplementation altered selected intestinal antioxidant-related parameters in juvenile silver pomfret (Table 8). The activities of CAT, GPX, and SOD were significantly elevated in the intestine of the inulin-fed group compared with the control group. The inulin-fed group also exhibited higher T-AOC than the control group. In contrast, no significant difference was observed in MDA levels between the two groups. Collectively, these results indicate that dietary inulin supplementation affected selected intestinal antioxidant-related indicators in juvenile silver pomfret.

### 3.8. Dietary Inulin Enhances Intestinal Digestive Enzyme Activities in Silver Pomfret

The effects of dietary inulin supplementation on intestinal digestive enzyme activities in juvenile silver pomfret are shown in Table 9. Dietary inulin supplementation altered selected intestinal digestive enzyme activities in juvenile silver pomfret. The activity of α-amylase was significantly higher in the inulin group. Similarly, the activity of lipase was also elevated in fish fed the inulin-supplemented diet. In contrast, the activity of pepsin did not differ between the dietary groups.

### 3.9. Dietary Inulin Improves Intestinal Morphology in Silver Pomfret

**Figure 2:**
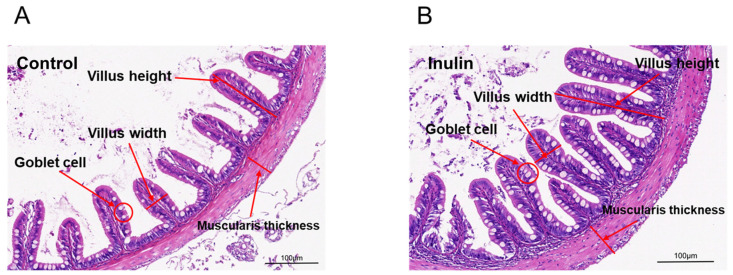
Dietary inulin improves intestinal morphology in juvenile silver pomfret. (**A**) Control, (**B**) Inulin groups (H&E staining, scale bar = 100 μm).

The effects of dietary inulin supplementation on intestinal histological parameters in juvenile silver pomfret are shown in Figure 2 and Table 10. Compared with the control group, the inulin group exhibited higher intestinal villus height and width. Additionally, inulin supplementation was associated with increased muscularis thickness. The number of goblet cells was also higher in the inulin group. Collectively, these results indicate that dietary inulin supplementation altered intestinal histological parameters in juvenile silver pomfret.

### 3.10. Dietary Inulin Improves Intestinal Function and Barrier Integrity in Silver Pomfret

Dietary inulin supplementation altered the intestinal mRNA expression of genes associated with tight junction function in young silver pomfret (Table 11). Compared with the control group, the inulin group showed significantly increased mRNA expression of tight junction proteins (TJPs, including *Occludin*, *ZO-1* and *Claudin-4*). No significant difference was observed in *Zonula Occludens-3* (*ZO-3*) expression between groups. Notably, *Claudin-15a*, a pore-forming claudin associated with increased permeability, was significantly downregulated.

### 3.11. Dietary Inulin Supplementation Alters the Gut Microbial Community Structure in Silver Pomfret

**Figure 3:**
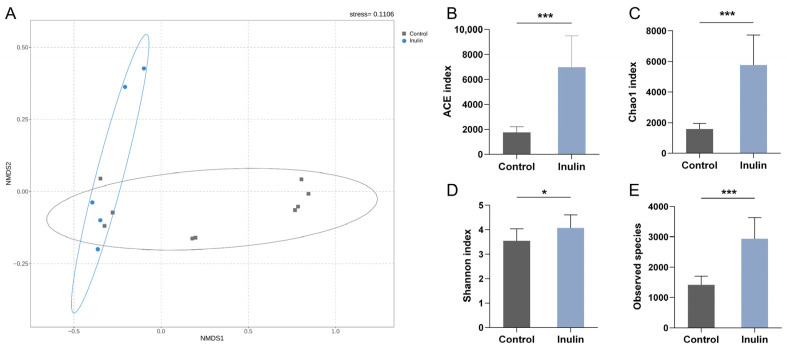
Dietary Inulin Supplementation Alters the Gut Microbial Community Structure in Silver Pomfret (**A**) Non-metric Multidimensional Scaling (NMDS) plot illustrating microbial community differences between groups (stress = 0.1106 < 0.2, indicating a reliable representation). Comparison of (**B**) ACE, (**C**) Chao1, (**D**) Shannon, and (**E**) observed species. Values are presented as mean ± SD (*n* = 3 replicate tanks per treatment). Asterisks indicate significant differences (* *p* < 0.05, *** *p* < 0.001).

The gut microbiota is closely associated with growth, nutrient metabolism, and immune function in juvenile silver pomfret. Dietary inulin supplementation altered the gut microbial community structure, as revealed by 16S rRNA sequencing. Alpha diversity indices, including ACE, Chao1, and Shannon, were higher in the inulin group (Figure 3B–E), indicating increased microbial richness and diversity. NMDS analysis showed a distinct separation between the inulin and control groups, reflecting differences in overall microbial community composition (Figure 3A). At the phylum level, the inulin group showed a lower relative abundance of *Proteobacteria*, a phylum that includes many taxa previously reported as opportunistic bacterial members, while increasing the relative abundance of *Bacteroidetes*, whose members are often associated with carbohydrate degradation and fiber utilization (Table 12). At the family level, inulin supplementation increased the relative abundance of *Chitinophagaceae* and *Bacillaceae*, which have been associated with chitin degradation and probiotic potential in previous studies, respectively, while *Vibrionaceae*, a family containing several opportunistic bacterial taxa in aquatic animals, showed a lower relative abundance (Table 12). At the genus and species levels, dietary inulin supplementation showed lower relative abundances of *Photobacterium* and *Photobacterium damselae* subsp. *damselae* (Table 12). Collectively, these results indicate that dietary inulin supplementation was associated with changes in the gut microbial community structure of juvenile silver pomfret.

## 4. Discussion

Juvenile silver pomfret aquaculture faces critical challenges, including immature intestinal function, dysbiotic microbiota, and compromised barrier integrity, which contribute to growth retardation and high mortality. Here, we found that dietary inulin supplementation was associated with improvements in intestinal homeostasis and systemic health status through changes in barrier-related, microbial, and immune-related responses. Specifically, inulin improved growth performance (e.g., increased WGR and SGR), modified selected nutrient-related indicators (e.g., elevated essential amino acids and MUFAs), enhanced intestinal morphology (e.g., villus height and muscularis thickness), and reshaped gut microbiota by altering the abundance of taxa previously associated with fiber utilization, probiotic potential, or opportunistic bacterial groups. These findings suggest that dietary inulin supplementation was associated with favorable changes in growth performance, digestive enzyme activities, immune-related and antioxidant-related indicators, and selected muscle compositional traits under the present experimental conditions. Critically, these changes were also accompanied by improvements in selected muscle compositional traits, particularly amino acid composition and selected fatty acid components.

In the present study, dietary inulin promoted growth performance via multifaceted optimization of intestinal function in juvenile silver pomfret. Our data reveal that inulin supplementation increased key growth metrics, including WGR and SGR, and decreased FCR (Table 3). The improved growth performance may be associated with changes in intestinal morphology and digestive enzyme activities. Specifically, inulin significantly enhanced the activities of pivotal digestive enzymes (α-amylase and lipase) (Table 9), potentially facilitating digestion of carbohydrates and lipids [26,27]. Inulin is a typical fructan composed predominantly of β-(2 → 1)-linked fructosyl units (Figure 1E–H), a structural feature that renders it resistant to digestion by host enzymes while making it highly fermentable by intestinal microbiota [28,29]. Furthermore, the porous and loose structure of inulin may enhance microbial accessibility by increasing its specific surface area, thereby promoting microbial fermentation (Figure 1A–C). This unique configuration allows inulin to reach the distal intestine intact, where it may act as a fermentable substrate for intestinal microbes. Therefore, SCFA-related processes may be a possible contributor to the observed intestinal responses, but this mechanism remains to be verified in silver pomfret. Previous studies have suggested that SCFA-associated luminal acidification may influence digestive enzyme activity and nutrient hydrolysis [30]. These findings align with prior studies on grass carp (*Ctenopharyngodon idella*) and Nile tilapia (*Oreochromis niloticus*), where inulin similarly enhanced digestive enzyme activities [31,32]. Histological examinations further revealed that inulin administration significantly increased intestinal villus height and muscularis thickness (Figure 2 and Table 10). The expanded villus surface area may improve nutrient absorption capacity, while the thickened muscle layer likely enhances intestinal peristalsis, which could facilitate nutrient uptake efficiency, ensuring a more sufficient supply of substrates for growth and muscle deposition [33,34]. Moreover, increased muscularis thickness also serves as a reliable indicator of intestinal maturation and is functionally linked to reinforced barrier protection. These morphological improvements in juvenile silver pomfret are consistent with observations in Japanese eel (*Anguilla japonica*) [35], golden pompano (*Trachinotus ovatus*) [36], and hybrid catfish [37], suggesting a conserved role of prebiotics in piscine intestinal development. Collectively, our findings indicate that intestinal development is critical for juvenile silver pomfret aquaculture, and dietary inulin supplementation may help relieve physiological constraints in intestinal function, thereby serving as a potential strategy to improve growth performance, survival rates, and production quality. These physiological improvements may partly explain the observed changes in muscle nutrient composition under the present experimental conditions.

Beyond the growth-enhancing effects, an important finding from a food science perspective is that dietary inulin modified selected muscle compositional traits in juvenile silver pomfret by modulating amino acid composition, especially essential amino acid profiles and flavor-related amino acids, and selected fatty acid components, thereby contributing to changes in the nutritional composition of silver pomfret as a food product. Specifically, inulin increased EAAs such as Met, Thr, and His (Table 4). Notably, as limiting amino acids in aquafeeds, elevated Met and Thr levels may support protein synthesis and anabolic processes, aligning with the observed improvements in efficient nutrient utilization and growth performance [38,39,40]. These amino acids also contribute to muscle protein deposition and nutritional composition in fish [39,40]. Concurrently, dietary inulin enriched key flavor-related amino acids, notably umami-imparting glutamate and sweetness-contributing glycine and serine (Table 4). This change in AA composition parallels findings in ducks, where inulin similarly increased umami and sweet AAs while reducing bitter compounds, suggesting a possible role of inulin in modulating flavor-related amino acid profiles [41]. These findings are consistent with previous reports in other animals, suggesting that dietary inulin may influence muscle amino acid profiles. However, whether such compositional changes translate into perceptible sensory differences in silver pomfret requires further verification. Among these, the elevation of Gly and Ser is particularly noteworthy, as these AAs serve as essential precursors for glutathione synthesis, suggesting a potential mechanism by which inulin may bolster the antioxidant defense system in muscle [42]. This aligns with the broader role of inulin in influencing nutritional and physiological traits, such as oxidative stress resilience. In parallel, inulin induced changes in fatty acid composition, characterized by reduced selected SFAs (e.g., C14:0, C15:0, C17:0) and elevated MUFAs, particularly oleic acid (C18:1n-9) (Table 5). These changes suggest that inulin supplementation may influence selected fatty acid components in silver pomfret muscle. MUFAs are important components of cell membranes and may contribute to membrane fluidity, nutrient transport, and energy metabolism [43,44,45]. Therefore, the increased MUFA levels observed in the inulin group may be related to improved physiological adaptability and nutrient utilization. In addition, oleic acid has been associated with cardiovascular health benefits in humans, including a reduced risk of atherosclerosis and improved cardiac function [46,47]. However, because total PUFA, EPA, and DHA were not significantly changed in the present study, the fatty acid results should be interpreted as a modification of selected fatty acid components rather than as a comprehensive improvement in lipid nutritional quality. Similar selective responses of muscle fatty acid profiles have been reported in previous fish nutrition studies, where dietary interventions altered certain fatty acids or fatty acid classes without consistently improving EPA, DHA, EPA+DHA, or total PUFA levels [48,49]. These changes in muscle composition may be driven by inulin-mediated enhancement of intestinal health, including elevated digestive enzyme activity and improved intestinal morphology, which may facilitate nutrient assimilation and subsequent availability of dietary precursors for muscle deposition. Collectively, this study underscores that early-life inulin intervention may serve as a nutritional approach with dual relevance to juvenile fish health and selected muscle compositional traits. Although direct sensory evaluation was not performed, the present results suggest that dietary supplementation with 5 g/kg inulin may improve selected aspects of muscle nutritional composition in young silver pomfret, particularly amino acid composition and certain fatty acid components.

The intestine serves not only as the primary site for nutrient digestion but also as a critical mucosal immune barrier, particularly in juvenile silver pomfret. However, its functional immaturity, characterized by underdeveloped structure, weak tight junctions, high intestinal permeability, and unstable gut microbiota composition, compromises both nutrient absorption and immune defense, thereby heightening susceptibility to infections and environmental stressors [50,51]. Our results indicate that dietary inulin supplementation improved several intestinal health-related indicators through changes in morphology, barrier integrity, and the microbial community, thereby partly alleviating these physiological vulnerabilities of young fish (Table 10, Figure 2 and Figure 3). First, inulin improved intestinal morphology by increasing villus height and width, and muscularis thickness (Table 10 and Figure 2). The increase in goblet cell count in the inulin-fed group may further support the mucosal barrier by enhancing mucus secretion and mucosal immune protection, which could contribute to intestinal protection against external challenges [52,53]. Secondly, in our present study, inulin was associated with improved intestinal barrier integrity by upregulating TJPs (such as *Occludin*, *ZO-1*, and *Claudin-4*), while downregulating the pore-forming *Claudin-15a* (a gene associated with increased intestinal permeability) (Table 11), thereby potentially reducing paracellular permeability and pathogen infiltration risks [54,55,56]. These findings are consistent with previous research in the intestines of grass carp, Japanese eel (*Anguilla japonica*) [57], golden pompano (*Trachinotus ovatus*) [57], and hybrid catfish [37], showing that inulin enhances *Occludin* expression and promotes intestinal development, highlighting the role of inulin in supporting intestinal tight junctions [58]. The improved intestinal barrier in inulin-fed juvenile silver pomfret likely contributed to the observed systemic immune-related changes, as an intact gut lining can limit microbial translocation and support immune regulation [12,59]. This was evidenced by increases in key innate immune parameters, specifically serum LZM and acid ACP activities (Table 6), indicating enhanced innate immune-related responses. The gut microbiota serves as a critical regulator of host health, influencing growth, nutrient metabolism, and immune responses in fish [26]. Our results reveal that dietary inulin altered the gut microbial community in juvenile silver pomfret, suggesting a shift in community structure. Specifically, inulin enhanced alpha diversity indices (ACE, Chao1, and Shannon), indicating increased microbial richness and diversity (Figure 3B–D). NMDS analysis showed a clear separation between the inulin and control groups (Figure 3A), reflecting differences in overall community composition. Beyond modulating overall community structure, inulin induced taxonomic shifts in the gut microbiota at multiple levels. At the phylum level, inulin reduced the abundance of *Proteobacteria*, a phylum that includes many opportunistic bacterial members, while increasing *Bacteroidetes*, members of which are often associated with carbohydrate degradation and fiber utilization. *Bacteroidetes* may participate in the fermentation of indigestible fibers and SCFA production according to previous studies (Table 12) [60,61]. At the family level, inulin increased the relative abundance of *Chitinophagaceae,* which has been associated with chitin degradation, and *Bacillaceae*, which includes taxa with reported probiotic potential (Table 12) [62,63,64,65]. Conversely, the abundance of *Vibrionaceae*, a family harboring potential pathogens like Vibrio and *Photobacterium*, was significantly suppressed (Table 12). Most notably, at the genus level, inulin significantly reduced the abundance of *Photobacterium*, particularly PDD (Table 12). The reduction in this primary pathogen of silver pomfret [7] may be partly related to changes in the gut microbial community structure, including possible competitive interactions among microbial taxa. Collectively, these modifications suggest that inulin supplementation reshaped the intestinal microbial community in a direction potentially favorable for gut homeostasis.

The beneficial effects of dietary inulin extended beyond the intestinal tract, eliciting a coordinated enhancement of systemic physiological resilience by optimizing the serum metabolic profile and bolstering innate immunity in juvenile silver pomfret. Inulin supplementation optimized the serum biochemical indicators, elevating serum glucose levels and reducing triglyceride concentrations, which may indicate changes in energy and lipid metabolism (Table 6). Concurrently, antioxidant-related enzyme activities increased, as reflected by higher activities of T-AOC, CAT, and GPX in both liver and intestine (Table 7 and Table 8), consistent with the role of inulin in upregulating antioxidative pathways in grass carp [58]. This upregulation suggests that dietary inulin may contribute to improved antioxidant status and physiological resilience. Previous studies have reported that inulin-derived microbial metabolites, especially butyrate, can modulate cytoprotective pathways such as the Nrf2-Keap1 pathway and antioxidant gene expression [66]. Concurrently, inulin strengthened innate immunity by elevating serum LZM and ACP activities (Table 6), which are critical for pathogen clearance and phagocytic function [67]. The enhanced antioxidant status may reduce mucosal oxidative damage, while the improved immune-related indicators (Table 6) may contribute to maintaining intestinal immune homeostasis, collectively reinforcing gut barrier integrity via the upregulation of TJPs (such as *Occludin*, *ZO-1*, and *Claudin-4*) (Table 11). Furthermore, inulin altered the gut microbial community, including an increase in *Bacteroidetes* (Table 12). Members of *Bacteroidetes* have been reported to participate in carbohydrate degradation and SCFA production [68]. This intestinal microbial shift may help maintain mucosal homeostasis and reduce physiological stress, thereby potentially lowering systemic inflammatory and metabolic burden. Collectively, these changes, involving intestinal modulation as well as systemic metabolic, antioxidant, and immune-related responses, suggest a more favorable physiological status in juvenile silver pomfret under the present experimental conditions. From a food science and technology standpoint, such a physiological status may be relevant to nutrient utilization and growth. It contributes to enhanced growth and selected muscle compositional changes. Consequently, early-life dietary inulin intervention should be interpreted as a gut-targeted nutritional approach rather than as definitive nutritional programming. It may help address a key production bottleneck in sustainable aquaculture and improve selected muscle nutritional compositional traits of silver pomfret under the present experimental conditions.

Overall, the present study supports the potential of dietary supplementation with 5 g/kg inulin as a gut-targeted nutritional strategy for young silver pomfret, with positive effects on intestinal health-related indicators and selected muscle nutritional traits. Because the response to prebiotics may vary with inclusion level and developmental stage, future studies using graded inulin levels would help refine its practical application. Further integration of microbial metabolite profiling, sensory and texture assessments, and targeted functional validation would provide deeper insight into how dietary inulin contributes to gut homeostasis and muscle nutritional quality in young silver pomfret.

## 5. Conclusions

This study showed that dietary supplementation with 5 g/kg inulin for 8 weeks was associated with improved growth performance, enhanced digestive enzyme activities, improved intestinal histological parameters, altered intestinal barrier-related gene expression, and changes in the gut microbial community structure of juvenile silver pomfret. In addition, inulin supplementation was accompanied by changes in selected muscle compositional traits, including increased levels of several essential and flavor-related amino acids and altered selected fatty acid components, mainly reflected by reduced levels of several saturated fatty acids and increased MUFA levels. These findings suggest that dietary inulin may serve as a potential gut-targeted nutritional approach for improving selected intestinal health-related indicators and muscle nutritional traits in juvenile silver pomfret.

## Figures and Tables

**Table 1:** Ingredient composition and proximate nutritional composition of the experimental diets.

Ingredients	Treatments	
	Control	Inulin
Final experimental diet composition, %		
Commercial compound feed	100.0	99.5
Inulin	0	0.5
Main ingredient composition of the basal commercial compound feed, % of commercial feed		
Fish meal	55	55
Krill meal	5	5
Squid meal	5	5
Shrimp meal	15	15
Potato starch	8	8
Shrimp oil	1	1
Squid oil	1	1
Beer yeast powder	2	2
Soybean lecithin oil powder	3	3
Vitamin premix ^1^	2	2
Mineral premix ^2^	1	1
Other additives ^3^	2	2
Proximate nutritional composition of final experimental diets, %		
Crude protein	52.6	52.4
Crude fat	8.7	8.4
Crude ash	17.4	16.8
Calcium	≥2	≥2
Total phosphorus	≥1.5	≥1.5
Crude fiber	≤5	≤5
Amino acid (lysine)	≥1	≥1

Vitamin premix ^1^: vitamin A, vitamin D3, dl-α-tocopherol (vitamin E), vitamin B1, vitamin B2, vitamin B6, vitamin B12, vitamin K3, niacin, D-calcium pantothenate, folic acid, L-ascorbic acid (vitamin C), D-biotin. Mineral premix ^2^: zinc methionine complex, manganese methionine complex, iron methionine complex, copper methionine complex, calcium iodate, cobalt sulfate, disodium hydrogen phosphate, calcium lactate. Other additives ^3^: benzoic acid, taurine, sucrose fatty acid esters, ethoxyquin, butylated hydroxyanisole. Although the two diets were broadly comparable in proximate composition, the inulin-supplemented diet was prepared by replacing 0.5% of the commercial feed with inulin, and no inert fiber or carbohydrate control was included.

**Table 2:** Primers for quantitative real-time polymerase chain reaction (qRT-PCR) analysis.

Gene	Primer Sequence (5′-3′)	Amplicon Size (bp)	R^2^	Amplification Efficiency (%)
*Occludin*	F (5′ → 3′) AACTATGGTTACGGCTCACTTGG	146	0.996	108.1
	R (5′ → 3′) CTGCTCTGTGATAGGCTCTGGT			
*ZO-1*	F (5′ → 3′) GAGTCTCTCCTTTCTCCTTGTC	226	0.994	93.6
	R (5′ → 3′) AGTCCCCTGGACGATGA			
*ZO-3*	F (5′ → 3′) TGGGAGCAACATACAATAACGC	138	0.993	104.8
	R (5′ → 3′) TGGTCCATTTGGCAACACG			
*Claudin-4*	F (5′ → 3′) CATAGGCTGGGCCTCTTCTG	141	0.993	100.0
	R (5′ → 3′) GACATGCGGGATGAATAGTCG			
*Claudin-15a*	F (5′ → 3′) ATCCGATTGTGGAAGTAGTGGC	103	0.996	104.5
	R (5′ → 3′) GTAACCGTTCAAAGCGAGCAG			
*β-actin*	F (5′ → 3′) TGGCATCACACCTTCTACAAC	157	0.992	106.2
	R (5′ → 3′) ACGACCAGAGGCATACAGG			

F, forward primer; R, reverse primer. R^2^ and amplification efficiency were calculated from standard curves generated using serially diluted cDNA. The specificity of each primer pair was verified by melting curve analysis. β-actin was used as the reference gene for normalization.

**Table 3:** Dietary Inulin Supplementation Enhances Growth Performance in Silver Pomfret.

Items	Treatments		*p*-Value
	Control	Inulin	
**Final weight, g**	19.19 ± 1.26 ^a^	22.50 ± 1.02 ^b^	0.0053
**WGR, %**	1353.79 ± 7.63 ^a^	1604.45 ± 6.18 ^b^	0.0053
**SGR, %/d**	4.72 ± 0.48 ^a^	5.02 ± 0.40 ^b^	0.0031
**FCR**	1.14 ± 0.04 ^a^	0.92 ± 0.18 ^b^	0.0011
**CF**	4.29 ± 0.32 ^a^	4.74 ± 0.41 ^b^	<0.001
**HSI, %**	1.12 ± 0.16	1.04 ± 0.21	0.1179

Values in the same row with different superscript letters indicate significant differences between groups (*p* < 0.05). Values are means ± SD (*n* = 3 replicate tanks per treatment).

**Table 4:** Dietary Inulin Alters Selected Muscle Amino Acid Profile in Silver Pomfret.

Items	Treatments		*p*-Value
	Control	Inulin	
Met, g/100 g wet weight	0.39 ± 0.01 ^a^	0.42 ± 0.02 ^b^	0.0340
Thr, g/100 g wet weight	0.69 ± 0.01 ^a^	0.72 ± 0.01 ^b^	0.0210
His, g/100 g wet weight	0.35 ± 0.02 ^a^	0.38 ± 0.01 ^b^	0.0340
TEAA, g/100 g wet weight	7.02 ± 0.21 ^a^	7.27 ± 0.06 ^b^	0.0330
Glu #, g/100 g wet weight	2.42 ± 0.03 ^a^	2.54 ± 0.03 ^b^	0.0080
Gly #, g/100 g wet weight	0.71 ± 0.02 ^a^	0.77 ± 0.02 ^b^	0.0120
Ser, g/100 g wet weight	0.64 ± 0.01 ^a^	0.67 ± 0.01 ^b^	0.0210
TAA, g/100 g wet weight	14.22 ± 0.21 ^a^	14.8 ± 0.06 ^b^	0.0110
Arg, g/100 g wet weight	0.91 ± 0.02 ^a^	0.95 ± 0.01 ^b^	0.0147
Ile, g/100 g wet weight	0.75 ± 0.01	0.77 ± 0.02	0.1890
Leu, g/100 g wet weight	1.26 ± 0.02	1.29 ± 0.02	0.1161
Lys, g/100 g wet weight	1.33 ± 0.02	1.36 ± 0.03	0.1890
Val, g/100 g wet weight	0.78 ± 0.02	0.8 ± 0.02	0.3453
Phe #, g/100 g wet weight	0.57 ± 0.01	0.59 ± 0.01	0.0705
Asp #, g/100 g wet weight	1.55 ± 0.03	1.59 ± 0.02	0.1234
Ala #, g/100 g wet weight	0.92 ± 0.02	0.95 ± 0.02	0.1401
Tyr #, g/100 g wet weight	0.43 ± 0.01	0.44 ± 0.01	0.2051
Pro, g/100 g wet weight	0.53 ± 0.01	0.56 ± 0.02	0.0808
EAA/TAA (%)	49.37 ± 0.04	49.15 ± 0.38	0.2888

Values in the same row with different superscript letters indicate significant differences between groups (*p* < 0.05). Data are expressed as g/100 g wet weight of dorsal muscle. Values are presented as mean ± SD (*n* = 3 replicate tanks per treatment). # Represents flavor-related amino acids; EAA/TAA: The ratio of essential amino acids to total amino acids.

**Table 5:** Dietary Inulin Supplementation Alters Selected Muscle Fatty Acid Composition in Silver Pomfret.

Items	Treatments		*p*-Value
	Control	Inulin	
**C14:0, %**	6.94 ± 0.08 ^a^	5.89 ± 0.63 ^b^	0.0460
**C15:0, %**	0.44 ± 0.01 ^a^	0.37 ± 0.03 ^b^	0.0220
**C17:0, %**	0.76 ± 0.01 ^a^	0.62 ± 0.06 ^b^	0.0230
**C17:1, %**	0.88 ± 0.06 ^a^	0.98 ± 0.01 ^b^	0.0430
**C18:1n-9, %**	30.32 ± 0.12 ^a^	32.75 ± 1.34 ^b^	0.0360
**MUFAs, %**	39.82 ± 0.14 ^a^	41.57 ± 0.67 ^b^	0.0110
**C18:2n-6T, %**	0.3 ± 0.01	0.32 ± 0.01	0.0532
**C18:0, %**	4.62 ± 0.05	4.15 ± 0.62	0.2658
**C20:0, %**	2.44 ± 0.02	2.46 ± 0.51	0.2420
**C14:1, %**	0.23 ± 0.01	0.2 ± 0.02	0.0647
**C16:1, %**	6.95 ± 0.04	6.17 ± 0.93	0.2239
**C20:1, %**	0.19 ± 0.02	0.16 ± 0.03	0.2420
**C22:1, %**	0.62 ± 0.01	0.76 ± 0.21	0.3203
**C24:1, %**	0.53 ± 0.04	0.64 ± 0.14	0.2843
**C18:2n-6C, %**	3.26 ± 0.09	2.58 ± 0.44	0.0620
**C18:3n-3, %**	0.71 ± 0.01	0.67 ± 0.09	0.4360
**C20:2, %**	0.19 ± 0.01	0.16 ± 0.05	0.3837
**C20:3, %**	0.14 ± 0.02	0.16 ± 0.03	0.4169
**C20:4, %**	0.63 ± 0.01	0.6 ± 0.03	0.1890
**C20:5, %**	4.95 ± 0.18	5.85 ± 1.63	0.3945
**C22:6, %**	8.57 ± 0.12	9.84 ± 2.92	0.4948
**PUFA**	18.75 ± 0.38	20.19 ± 4.04	0.5722

Values in the same row with different superscript letters indicate significant differences between groups (*p* < 0.05). Fatty acid contents are expressed as relative percentages of total fatty acids. Values are presented as mean ± SD (*n* = 3 replicate tanks per treatment).

**Table 6:** Dietary Inulin Improves Serum Metabolism and Enhances Innate Immunity in Juvenile Silver Pomfret.

Items	Treatments		*p*-Value
	Control	Inulin	
**TG, mmol/L**	2.15 ± 0.29 ^a^	1.43 ± 0.21 ^b^	0.0071
**GLU, mmol/L**	2.35 ± 0.28 ^a^	3.46 ± 0.37 ^b^	0.0032
**LZM, μg/mL**	4.80 ± 0.31 ^a^	9.44 ± 0.76 ^b^	0.0056
**ACP, U/L**	44.33 ± 1.85 ^a^	73.74 ± 1.99 ^b^	0.0055
**T-CHO, mmol/L**	6.08 ± 0.94	5.16 ± 0.35	0.3838

Values in the same row with different superscript letters indicate significant differences between groups (*p* < 0.05). Values are presented as mean ± SD (*n* = 3 replicate tanks per treatment).

**Table 7:** Dietary Inulin Enhances Liver Antioxidant Capacity in Silver Pomfret.

Items	Treatments		*p*-Value
	Control	Inulin	
**T-AOC, mmol/L**	0.51 ± 0.03 ^a^	0.58 ± 0.07 ^b^	0.0394
**CAT, U/mg prot**	83.61 ± 3.21 ^a^	112.80 ± 1.41 ^b^	0.0019
**GPX, nmol/min/g**	288.50 ± 1.11 ^a^	365.10 ± 1.65 ^b^	<0.001
**SOD, U/mg prot**	11.25 ± 1.25	11.66 ± 0.78	0.7899
**MDA, mmol/g**	18.35 ± 1.56	20.53 ± 1.77	0.2447

Values in the same row with different superscript letters indicate significant differences between groups (*p* < 0.05). Values are presented as mean ± SD (*n* = 3 replicate tanks per treatment).

**Table 8:** Dietary Inulin Enhances Intestinal Antioxidant Capacity in Silver Pomfret.

Items	Treatments		*p*-Value
	Control	Inulin	
**T-AOC, mmol/L**	0.27 ± 0.33 ^a^	0.36 ± 0.05 ^b^	0.0051
**CAT, U/mg prot**	16.39 ± 4.32 ^a^	23.56 ± 2.79 ^b^	0.0066
**GPX, nmol/min/g wet weight**	202.80 ± 20.60 ^a^	306.50 ± 29.80 ^b^	0.0069
**SOD, U/mg prot**	5.26 ± 1.42 ^a^	9.15 ± 1.66 ^b^	0.0014
**MDA, nmol/g wet weight**	54.59 ± 2.80	45.90 ± 3.40	0.2158

Values in the same row with different superscript letters indicate significant differences between groups (*p* < 0.05). Values are presented as mean ± SD (*n* = 3 replicate tanks per treatment).

**Table 9:** Dietary Inulin Enhances Intestinal Digestive Enzyme Activities in Silver Pomfret.

Items	Treatments		*p*-Value
	Control	Inulin	
**α-amylase** **, U/mg prot**	0.42 ± 0.89 ^a^	0.67 ± 0.15 ^b^	0.0061
**lipase** **, nmol/min/g**	839.00 ± 17.10 ^a^	1151.00 ± 22.30 ^b^	0.0251
**pepsin, U/g**	196.80 ± 17.30	290.10 ± 23.10	0.1555

Values in the same row with different superscript letters indicate significant differences between groups (*p* < 0.05). Values are presented as mean ± SD (*n* = 3 replicate tanks per treatment).

**Table 10:** Dietary inulin improves intestinal morphology in juvenile silver pomfret.

Items	Treatments		*p*-Value
	Control	Inulin	
**villus height, µm**	147.10 ± 5.52 ^a^	211.90 ± 4.89 ^b^	<0.001
**villus width, µm**	52.61 ± 1.68 ^a^	63.15 ± 3.04 ^b^	<0.001
**muscularis thickness, µm**	55.15 ± 2.87 ^a^	69.84 ± 3.06 ^b^	<0.001
**goblet cells**	14.11 ± 2.89 ^a^	22.44 ± 2.01 ^b^	<0.001

Values in the same row with different superscript letters indicate significant differences between groups (*p* < 0.05). Values are presented as mean ± SD (*n* = 3 replicate tanks per treatment).

**Table 11:** Dietary Inulin Improves Intestinal Function and Barrier Integrity in Silver Pomfret.

Items	Treatments		*p*-Value
	Control	Inulin	
***Occludin***	1.00 ± 0.15 ^a^	1.81 ± 0.22 ^b^	<0.001
***ZO-1***	1.00 ± 0.06 ^a^	1.42 ± 0.06 ^b^	<0.001
***ZO-3***	1.01 ± 0.15	0.88 ± 0.04	0.1523
***Claudin-4***	1.08 ± 0.41 ^a^	6.57 ± 0.79 ^b^	<0.001
***Claudin-15a***	1.00 ± 0.07 ^a^	0.76 ± 0.29 ^b^	<0.001

Values in the same row with different superscript letters indicate significant differences between groups (*p* < 0.05). Values are presented as mean ± SD (*n* = 3 replicate tanks per treatment).

**Table 12:** Dietary Inulin Supplementation Alters the Gut Microbial Community Structure in Silver Pomfret.

Items	Treatments		*p*-Value
	Control	Inulin	
Proteobacteria, %	82.14 ± 2.11 ^a^	65.54 ± 2.23 ^b^	0.0019
Bacteroidetes, %	11.62 ± 1.13 ^a^	31.99 ± 1.05 ^b^	0.0012
Chitinophagaceae, %	11.39 ± 0.16 ^a^	31.75 ± 0.13 ^b^	0.0012
Bacillaceae, %	0.16 ± 0.02 ^a^	0.43 ± 0.01 ^b^	0.0075
Vibrionaceae, %	36.56 ± 1.62 ^a^	2.86 ± 0.44 ^b^	0.0061
Photobacterium, %	36.54 ± 1.23 ^a^	2.85 ± 0.25 ^b^	0.0061
*Photobacterium damselae*, %	30.47 ± 0.62 ^a^	2.39 ± 0.35 ^b^	0.0072
*Photobacterium damselae* subsp. *damselae*, %	6.07 ± 0.06 ^a^	0.46 ± 0.04 ^b^	0.0045

Values in the same row with different superscript letters indicate significant differences between groups (*p* < 0.05). Values are presented as mean ± SD (*n* = 3 replicate tanks per treatment).

## Data Availability

The original contributions presented in this study are included in the article/Supplementary Material. Further inquiries can be directed to the corresponding authors.

## Supplementary Materials

The following supporting information can be downloaded at: https://www.mdpi.com/article/10.3390/foods15132391/s1; Figure S1: Kaplan–Meier survival analysis of juvenile silver pomfret in the control and inulin-supplemented group over the 56-day experimental period.; Table S1: Expression stability of β-actin used as the reference gene for qRT-PCR normalization.

## Abbreviations

The following abbreviations are used in this manuscript:

SCFAs	Short-chain fatty acids
SEM	Scanning electron microscopy
NMR	Nuclear magnetic resonance
FTIR	Fourier-transform infrared spectroscopy
WGR	Weight gain rate
SGR	Specific growth rate
FCR	Feed conversion ratio
CF	Condition factor
HSI	Hepatosomatic index
SOD	Superoxide dismutase
T-AOC	Total antioxidant capacity
CAT	Catalase
GPX	Glutathione peroxidase
MDA	Malondialdehyde
GLU	Glucose
T-CHO	Total cholesterol
TG	Triglyceride
ACP	Acid phosphatase
LZM	Lysozyme
H&E	Hematoxylin and eosin
TEAA	Total essential amino acids
TAA	Total amino acids
FAMEs	Fatty acid methyl esters
PCR	Polymerase chain reaction
CCS	Circular consensus sequence
OTUs	Operational taxonomic units
RT-qPCR	Quantitative real-time PCR
SD	Standard deviation
Met	Methionine
Thr	Threonine
His	Histidine
Gly	Glycine
Ser	Serine
Arg	Arginine
Ile	Isoleucine
Leu	Leucine
Lys	Lysine
Val	Valine
Phe	Phenylalanine
Asp	Aspartic acid
Ala	Alanine
Tyr	Tyrosine
Pro	Proline
EAAs	Essential amino acids
SFAs	Saturated fatty acids
C14:0	Myristic acid
C15:0	Pentadecanoic acid
C17:0	Heptadecanoic acid
MUFAs	Monounsaturated fatty acids
C17:1	Heptadecenoic acid
C18:1n-9	Oleic acid
C18:0	Octadecanoic acid
C20:0	Eicosanoic acid
C14:1	Myristoleic acid
C16:1	Palmitoleic acid
C20:1	Eicosenoic acid
C22:1	Erucic acid
C24:1	Nervonic acid
C18:2n-6T	Linoleic acid (trans)
C18:2n-6C	Linoleic acid (cis)
C18:3n-3	α-Linolenic acid
C20:2	Eicosadienoic acid
C20:3	Dihomo-γ-linolenic acid
C20:4	Arachidonic acid
C20:5	Eicosapentaenoic acid
C22:6	Docosahexaenoic acid
PUFA	Polyunsaturated fatty acids
TJPs	Tight junction proteins
*ZO-3*	*Zonula Occludens-3*
*ZO-**1*	*Zonula Occludens-**1*
CAZymes	Carbohydrate-active enzymes
PDD	*P. damselae* subsp. *damselae*
