# Supplementary material for: Dietary Inulin Modulates Intestinal Health and Muscle Nutritional Composition in Juvenile Silver Pomfret (Pampus argenteus)
Source: Foods. 2026 Jul 5;15(13):2391. doi: 10.3390/foods15132391 (PMC13361336; doi:10.3390/foods15132391)
Supplement: Supplementary file 1 [file foods-15-02391-s001.zip › foods-4346089-supplementary.pdf]

Supplementary Materials

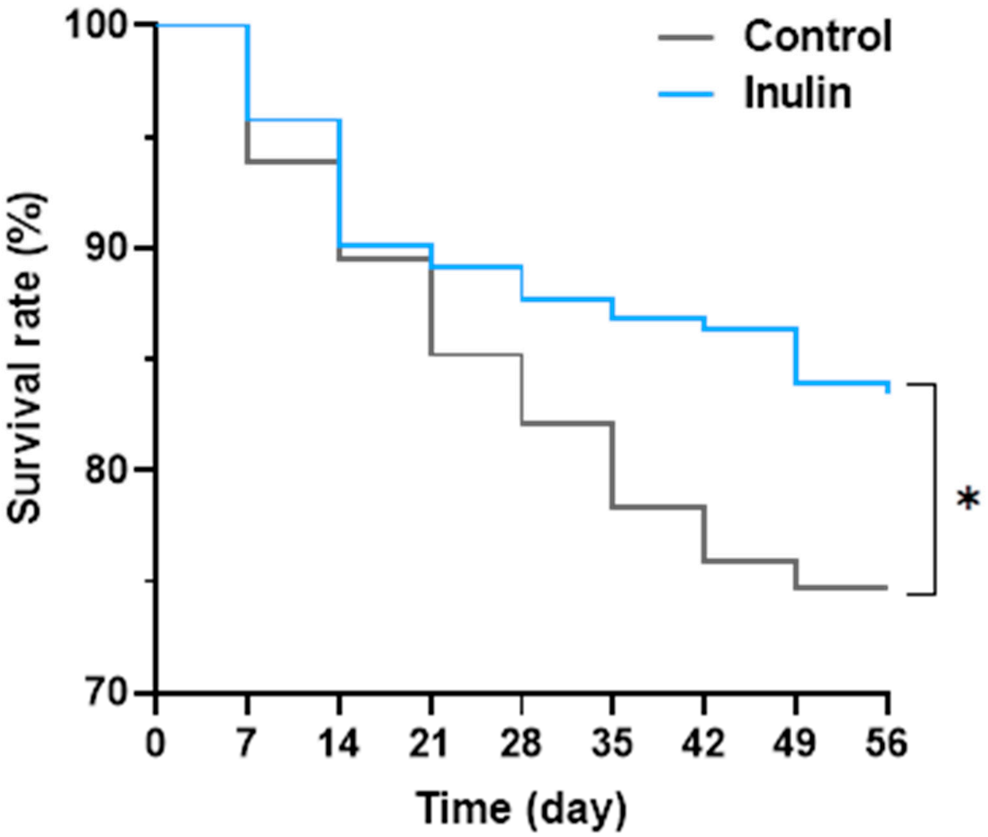

Figure S1. Kaplan–Meier survival analysis of juvenile silver pomfret in the control and inulin-supplemented group over the 56-day experimental period. (*n* = 3 replicate tanks per treatment).

Table S1. Expression stability of β-actin used as the reference gene for qRT-PCR normalization.

Reference gene	Control Mean Ct ± SD	Inulin Mean Ct ± SD	Control CV (%)	Inulin CV (%)	*P*-value
β-actin	16.48 ± 0.03	16.50 ± 0.06	0.172	0.335	0.774

Values are presented as mean Ct, SD, and coefficient of variation (CV). The stability of β-actin expression was evaluated by comparing Ct values between the control and inulin groups. No significant difference was observed between groups (P = 0.774), supporting the use of β-actin as the reference gene for qRT-PCR normalization.
